# A metal-aware library-expansion and virtual-screening workflow for *Pseudomonas aeruginosa* ATCC 15692 (PAO1) MraY identifies compound 5311309 with stable binding and mid-micromolar activity

**DOI:** 10.3389/fmicb.2026.1798540

**Published:** 2026-04-07

**Authors:** Tao Shen, Lichen Zhang, Rui Chen, Shengnan Jia, He Zhengfu

**Affiliations:** 1 Department of Thoracic Surgery, Sir Run Run Shaw Hospital, Zhejiang University School of Medicine, Hangzhou, China; 2 School of Bioengineering, Jiangnan University, Wuxi, China; 3 Department of General Surgery, Sir Run Run Shaw Hospital, Zhejiang University School of Medicine, Hangzhou, China

**Keywords:** 5311309, ADMET, Mg^2+^/metal site, MM-GBSA, molecular dynamics, MraY (MRAY_PSEAE), *Pseudomonas aeruginosa* ATCC 15692 (PAO1), structure-based drug design

## Abstract

**Introduction:**

*Pseudomonas aeruginosa* lung infections are difficult to eradicate because of biofilms and rising resistance. MraY (MRAY_PSEAE), an essential membrane enzyme in peptidoglycan biosynthesis, depends on divalent metals (notably Mg^2+^); therefore, metal coordination is critical for reliable structure-based prioritization.

**Methods:**

We built an explicit protein–ligand–metal model of MRAY_PSEAE and extracted pocket constraints from point-cloud geometry and polar/charged features. Candidates were generated through similarity-guided library expansion, combining PubChem 2D similarity retrieval with local rule-based BRICS enumeration. After canonicalization and 2D/property filtering, compounds were clustered by fingerprint similarity and prioritized through chemical-state plausibility, docking, Prime/MM-GBSA rescoring, and ligand strain filtering, followed by interaction analysis, 1-μs MD, and *in silico* ADMET.

**Results:**

Three hits (6675, 2733768, 5311309) were prioritized. In microsecond MD, compound 5311309 showed the most stable pose and key hotspot retention. ADMET indicated a permeability/efflux bottleneck along with liver–kidney and irritation/sensitization alerts, whereas hERG and AMES signals were favorable. In growth assays, compound 5311309 showed an IC50 of approximately 15.1 μM and an MIC of 64 μM.

**Conclusion:**

Metal-aware pocket modeling plus multi-level screening improved plausibility and ranking for this metal-dependent target, identifying compound 5311309 as a practical lead optimization starting point.

## 1 Introduction

Pseudomonas aeruginosa is a leading cause of severe and persistent lung infections, ranging from ventilator-associated pneumonia in hospitalized patients to chronic airway colonization in cystic fibrosis (CF) (Qin et al., 2022). A hallmark of *P. aeruginosa* pulmonary disease is its propensity to form multicellular biofilms, which restrict antibiotic penetration, promote tolerant subpopulations, and complicate immune clearance—collectively making eradication difficult and relapse common (Moreau-Marquis et al., 2008; Oluyombo et al., 2019). The therapeutic challenge is amplified by accelerating antimicrobial resistance (AMR) (Ferrara et al., 2024). In its 2024 update of the Bacterial Priority Pathogens List (BPPL), the World Health Organization (WHO) continues to highlight carbapenem-resistant *P. aeruginosa* as a high-priority AMR threat, underscoring the ongoing need for new anti-infectives and non-cross-resistant mechanisms. These realities motivate renewed efforts to identify essential bacterial targets that are distinct from those addressed by existing antibiotic classes and that can enable rational, structure-guided inhibitor discovery.

Bacterial cell-wall biogenesis—particularly peptidoglycan assembly—remains one of the most validated, selective, and clinically actionable antibacterial pathways (Torrens and Cava, 2024). Within this pathway, phospho-MurNAc-pentapeptide translocase MraY catalyzes the first membrane-committed step: transfer of the phospho-MurNAc-pentapeptide moiety from UDP-MurNAc-pentapeptide to the lipid carrier undecaprenyl phosphate (C55-P), producing lipid I, an indispensable precursor for downstream peptidoglycan synthesis (Liu et al., 2016). In the UniProt nomenclature for *P. aeruginosa*, this enzyme is often referred to as MRAY_PSEAE (e.g., associated with accession Q9HVZ8 in curated listings). Because MraY operates at the interface of cytosolic nucleotide chemistry and membrane-embedded lipid processing, its inhibition can collapse cell-wall construction at an early choke point, offering strong potential for bactericidal intervention.

Importantly, MraY catalysis and substrate handling are tightly coupled to divalent metal ions, most notably Mg^2+^, which participate in stabilizing highly charged phosphate groups during transfer chemistry (Cao et al., 2025). A mechanistic and chemical–biology synthesis of the field explicitly describes MraY as performing the C55-P transfer in the presence of an Mg^2+^ cofactor, linking metal coordination directly to productive catalysis and inhibitor binding energetics (Nakaya et al., 2022). Consistent with this finding, multiple classes of nucleoside natural products (e.g., muraymycins, mureidomycins, liposidomycins/caprazamycins, and tunicamycins) have validated MraY as druggable; however, translating these complex scaffolds into clinically viable agents remains challenging (Mashalidis et al., 2019). For Gram-negative pathogens such as *P. aeruginosa*, additional constraints—including outer-membrane penetration, efflux, and the need to maintain potency in the membrane-proximal catalytic site—further increase the challenegesacc for successful lead discovery (Saxena et al., 2023).

Computational structure-based drug design (SBDD) provides a scalable route to explore chemical space, but metal-containing active sites remain a recurring failure mode for conventional docking and scoring (Xu et al., 2024). Metalloprotein docking is frequently complicated by three-body interactions among residues, metal ions, and ligands, along with strict coordination geometry requirements that are not always captured by standard force fields or empirical scoring functions (Wang et al., 2020). The community response has included specialized metal-aware docking and modeling approaches, reflecting a broad recognition that explicit treatment of metal coordination can substantially improve pose fidelity and screening utility (Zhang et al., 2026). For MraY—where a Mg^2+^-stabilized, phosphate-rich catalytic environment is central—neglecting metal physics can lead to distorted binding hypotheses and unreliable rank ordering, ultimately undermining downstream synthesis prioritization.

In parallel, deep generative models have rapidly evolved from 2D *de novo* design tools into 3D, pocket-conditioned frameworks capable of proposing ligand geometries consistent with target binding sites. Comprehensive reviews have documented how VAEs, GANs, autoregressive transformers, and diffusion models can explore vast chemical space while optimizing multi-objective criteria relevant to drug discovery (Cheng et al., 2021). More recently, equivariant diffusion models have demonstrated practical structure-based generation conditioned directly on protein pockets, enabling iterative generate–score–refine workflows in 3D (Schneuing et al., 2024). However, for metal-dependent targets, naïve pocket conditioning is often insufficient: the model must learn—or be constrained to respect—the coordination number, preferred donor atom types, and geometry-dependent energetics that govern Mg^2+^-mediated recognition.

Against this backdrop, this study focuses on MRAY_PSEAE (MraY) as a high-value antibacterial target for *P. aeruginosa* ATCC 15692 (PAO1) and establishes a metal-aware, structure-based candidate discovery workflow rather than a learned *de novo* generative model. The pocket metal ion is treated as a first-class structural feature by explicitly encoding the Mg^2+^ position and its coordination context and by coupling candidate sourcing (PubChem similarity retrieval plus BRICS rule-based enumeration) with metal-sensitive filtering and scoring to maintain physically plausible binding hypotheses. This framework is intended to provide synthetically actionable MraY inhibitor candidates and to serve as a practical template for metal-informed structure-based screening.

## 2 Methods

### 2.1 Protein–ligand complex input and ligand identification

The reference complex model was obtained from AlphaFill (Q9HVZ8). In this run, the model MRAY_PSEAE was used. The bound ligand residue name was either specified manually or inferred automatically as the non-protein, non-water, non-metal residue with the largest number of heavy atoms (minimum five heavy atoms).

### 2.2 Pocket definition and metal ion handling

The binding pocket was defined as all standard amino-acid residues with any heavy atom within 6.0 Å of any ligand heavy atom. All pocket heavy-atom coordinates were retained as a point cloud for fast distance-based interaction counting. Pocket donor and acceptor point sets were assigned using lightweight, residue-aware heuristics (e.g., Lys/Arg/His N atoms as donors; Asp/Glu O atoms as acceptors; Ser/Thr/Tyr O atoms as donors/acceptors). These descriptors were used for interaction summarization and downstream ranking rather than as docking-grade hydrogen-bond constraints.

Metal ions were identified as single-atom (non-water and non-amino-acid) residues whose element belonged to a curated set (e.g., Zn, Mg, Fe, Mn, Cu, Co, Ni, Ca, Na, and K) and whose distance to the ligand was within max(cutoff + 2 Å, 6 Å), i.e., 8 Å for a 6 Å pocket cutoff. Each metal was assigned a nominal charge (e.g., Ni^2 +^ and Mg^2+^). In MRAY_PSEAE.pdb, three proximal metal ions (2 × Ni and 1 × Mg) were detected and retained during protein/receptor preparation, and the docking grid was generated with these ions present. Glide docking was then performed using standard settings, without additional metal-specific coordination constraints or custom metal-scoring terms (the screened ligands were metal-free organic molecules). Accordingly, “metal-aware” in this study refers to explicit metal-site representation in receptor preparation and downstream metal-aware scoring/analysis, rather than the use of a specialized Glide metal-coordination protocol.

### 2.3 PubChem similarity retrieval

If enabled, a 2D similarity search was performed via PubChem PUG REST (fastsimilarity_2d) using the reference SMILES, with a similarity threshold of 30/100 and a maximum of 1,000 records returned. Retrieved CIDs were converted to SMILES, canonicalized, filtered to single-fragment molecules, and deduplicated.

### 2.4 Local fragment-based enumeration (BRICS)

Two RDKit BRICS-based generators were applied. First, a single BRICS bond replacement can be achieved by substituting one fragment for an R-group library derived from diverse seed fragments. Second, BRICS recombination involves reassembling 2–3 fragments sampled from the reference BRICS fragments plus a small BRICS-labeled fragment pool. Candidates were sanitized, canonicalized, filtered into single-fragment molecules, and deduplicated.

### 2.5 Molecular similarity and property filtering

For each candidate SMILES, 2D similarity to the reference was computed as the maximum Tanimoto similarity across three fingerprints: Morgan fingerprint (radius 2, 2048 bits), RDKit topological fingerprint, and MACCS keys. Candidates with 2D similarity of < 0.02 were discarded.

Physicochemical properties were computed in RDKit, including molecular weight, logP, TPSA, HBD, HBA, rotatable bonds, ring count, and QED. Additional structural filters were applied: molecular weight 200–600 Da, logP −0.2–5.5, TPSA ≤ 160 Å2, minimum hetero atoms of ≥ 4, minimum hetero element types of ≥ 1, and a minimum functional-group count of ≥ 3 (functional groups were counted via SMARTS patterns). Lipinski rule-of-five violations were counted using standard thresholds (MW > 500, logP > 5, HBD > 5, HBA > 10); candidates with more than two rule-of-five violations were discarded. Candidates with QED < 0.30 were discarded.

### 2.6 3D conformer generation and pose alignment

For candidates passing the 2D and property filters, 3D conformers were generated with RDKit ETKDGv3 (random seed = 0), followed by UFF geometry optimization (up to 200 iterations). Candidate conformers were aligned to the bound reference ligand pose using RDKit alignment (AlignMol) with an atom mapping derived either from an exact substructure match (fast path) or from an MCS between the candidate and the reference; at least three mapped atoms were required. The aligned RMSD was recorded as an auxiliary output.

### 2.7 Sequence alignment and secondary structure annotation

When comparing homologous proteins, pairwise global alignment was performed using Biopython’s PairwiseAligner (match score = 1, mismatch score = −0.5, gap open penalty = −10, gap extension penalty = −0.5). Secondary structure was assigned from DSSP when available (mkdssp or dssp); otherwise, a phi/psi-based fallback classified residues into helix (H), strand (E), or coil (C). Alignment plots were rendered with Matplotlib and exported at 600 dpi in an ESPript-like style for manuscript figures.

### 2.8 Pose and protonation-state handling for docking

During docking, alternative ligand protonation/tautomeric states were handled using the Schrödinger state-penalty workflow, and multiple poses were sampled for each state. For downstream ranking, compounds with state penalty > 0 were excluded, and one representative best-ranked pose per retained ligand was propagated to MM-GBSA, ligand strain analysis, and MD stimulations.

### 2.9 Molecular dynamics simulations in a membrane environment

MD simulations were performed in Desmond 2025–1^1^ using the OPLS5 force field. Protein–ligand complexes were embedded in a POPC membrane, solvated with TIP3P water, neutralized, and set to 0.15 M KCl. After Desmond default membrane relaxation, 1.0 μs production runs were carried out in the NPT ensemble at 300 K and 1 bar (Nosé–Hoover thermostat; semi-isotropic MTK barostat), with PME electrostatics and a 9.0 Å short-range cutoff. Hydrogen-involving bonds were constrained using M-SHAKE, and trajectories were integrated with a 2-fs time step. No explicit distance/angle restraints were applied to enforce Mg^2+^/Ni^2+^ coordination. No explicit distance or angle restraints were applied to enforce Mg^2+^/Ni^2+^ coordination during production MD. Metal-associated interactions were inspected from trajectory interaction analyses; however, explicit metal–ligand distance/angle cutoffs were not used as hard selection criteria in the current study.

### 2.10 Concentration-dependent growth inhibition assay and IC50/MIC determination

Growth inhibition assays were performed using *Pseudomonas aeruginosa* ATCC 15692 (PAO1) in 96-well plates. Overnight cultures were diluted to a standardized starting inoculum and treated with a log-spaced concentration series at constant final dimethyl sulfoxide (DMSO; vehicle control), with five replicate wells per concentration. Endpoint OD_600_ was measured, background-corrected by subtracting the mean media-only blank signal, and then normalized to the blank-corrected vehicle control. High-concentration wells were visually inspected for precipitation; wells with visible precipitates were excluded from IC_50_ fitting. IC_50_ was obtained by four-parameter logistic fitting of log10 concentration–response data. Minimum inhibitory concentration (MIC) was defined as the lowest concentration with background-corrected OD_600_ of ≤ 0.05 and no detectable growth in all 5/5 replicates. This operational MIC readout was derived under our in-house assay conditions and does not represent a formal CLSI/EUCAST reference MIC determination.

## 3 Results and discussion

### 3.1 Structural characterization of the binding pocket and metal-site constraints for candidate screening

Motivated by the need to design metal-aware inhibitors for MRAY_PSEAE, we carried out multi-level characterization of the ligand-binding pocket to derive computable constraints for structure-conditioned generation and screening. We prioritized constraints that jointly capture spatial occupancy, polar and charged anchoring, and the local physicochemical environment surrounding the metal site, as these factors are expected to govern both posing feasibility and competitiveness against the reference binding mode.

**Figure 1:**
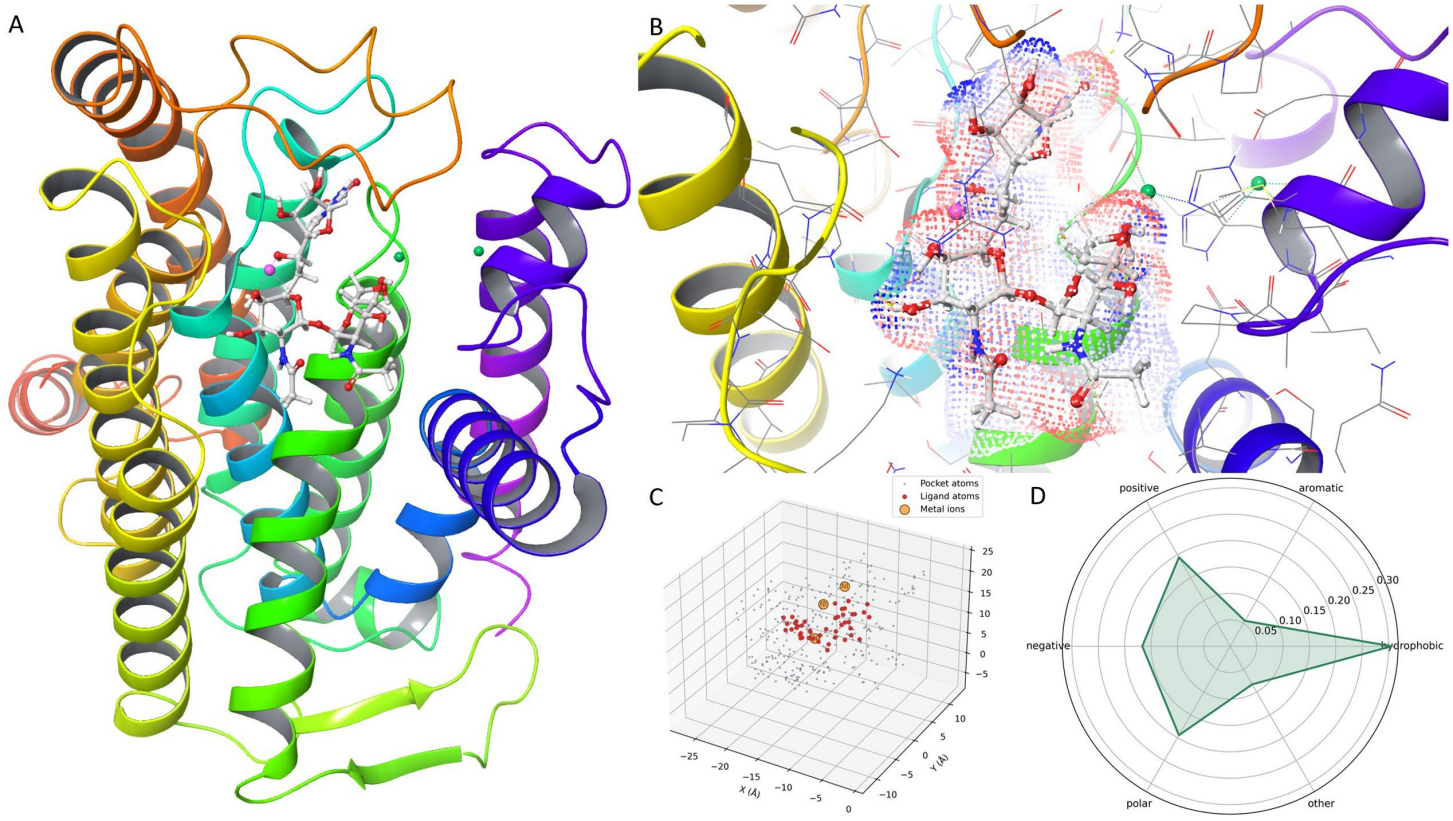
Multi-level characterization of the MRAY_PSEAE ligand binding pocket and metal site constraints. **(A)** Overall MRAY_PSEAE ligand metal ion complex model from AlphaFill. **(B)** Electron density map of the ligand in the pocket. **(C)** Pocket ligand point cloud with metal ions and interaction types. **(D)** Radar chart of pocket residue composition and ligand proximity.

We first constructed a protein–ligand–metal complex model for MRAY_PSEAE using AlphaFill (Figure 1A). AlphaFill enriches predicted protein models by transplanting missing ligands, cofactors, and metal ions from experimentally determined structures based on sequence and structural similarity, thereby providing a practical starting point that preserves the small-molecule and ion context for downstream modeling (Hekkelman et al., 2023). In the transferred model, both Mg^2+^ and Ni^2+^ were present. Consistent with the original MraY structural report (Chung, 2013 #582), we treated Mg^2+^ as the catalytically relevant divalent ion, whereas Ni^2+^ was considered crystallization-associated (loop E/HHH motif) and not a confirmed physiological cofactor. Building on this complex, we computed and visualized a ligand-associated electron density map within the pocket (Figure 1B). Electron density is routinely used in structural workflows to evaluate whether the data support ligand placement and to assess pose plausibility; thus, the density map provides an interpretable spatial prior for regions most compatible with ligand occupancy. A limitation is that explicit metal-coordination energetics were not parameterized during glide docking, which may reduce sensitivity to coordination-specific binding effects.

To simplify the pocket description while retaining interaction-critical information, we represented the binding site as a pocket-ligand point cloud with explicit inclusion of metal ions (Figure 1C). This representation encodes key interaction types as discrete points and is consistent with established pocket comparison and modern 3D pocket modeling practices that treat pockets as atom clouds or point clouds (Hoffmann et al., 2010). The pocket scale comprised 279 atomic points, 36 donor points, 41 acceptor points, and 54 hydrophobic points, indicating that the site requires substantial hydrogen-bond complementarity together with hydrophobic cavity filling. The transferred metal neighborhood contained one Mg^2+^ site and two Ni^2+^ sites. The nearest ligand-to-metal distance was 0.86 Å, indicating an extremely close metal neighborhood likely to impose strong geometric and electrostatic constraints; however, given the likely crystallization origin of Ni^2+^, we did not treat Ni^2+^-specific chelation as a physiological requirement during ranking. In practice, candidates were prioritized based on metal-proximal geometric feasibility and electrostatic complementarity centered on the Mg^2+^-relevant catalytic region, while poses that relied primarily on Ni^2+^-specific contacts were interpreted conservatively.

Finally, Figure 1D plots metal electrostatic similarity against total score and shows a near-zero correlation (Pearson *r* = −0.080). This likely reflects the narrow dynamic range of metal_electrostatic_sim relative to pocket_score, causing the metal term to act as a modest adjustment rather than a primary ranking driver. Accordingly, uncertainty in Ni™ assignment is unlikely to be a major determinant of final ranking in the current workflow. If metal coordination and electronic effects are intended to be decisive, discriminability and interpretability could be improved by increasing the weight of the metal term, introducing explicit coordination-geometry constraints (donor atom type, distance, and angle), and adopting a more rigorous electrostatic treatment at the metal site where feasible. Future studies will include sensitivity analyses comparing Mg-only and Mg + Ni receptor models, with explicit monitoring of coordination distances and angles.

### 3.2 Candidate sourcing, 2D similarity control, and multi-objective prioritization

To maintain practical chemical accessibility while preserving comparability to the reference ligand, the candidate set was sourced primarily through PubChem 2D similarity retrieval. This strategy intentionally focused exploration on near-neighbor chemistry likely to be purchasable or synthetically tractable while accepting reduced scaffold novelty. Because PubChem similarity is fingerprint-based, it captures 2D neighborhood relationships but does not guarantee compatibility with the 3D, metal-proximal MRAY_PSEAE pocket.

**Figure 2:**
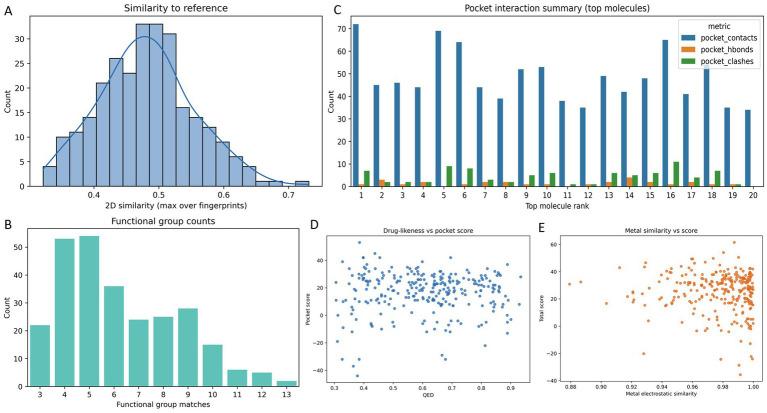
Candidate source similarity constraints and multi-objective selection signals. **(A)** Distribution of 2D similarity to the reference ligand. **(B)** Distribution of functional group SMARTS match counts and overall functional group composition. **(C)** Interaction summary of top 20 molecules with pocket contacts, pocket H-bonds, and pocket clashes. **(D)** QED versus pocket score and the Pareto frontier used for shortlist selection. **(E)** Metal electrostatic similarity versus total score.

As shown in Figure 2A, selected compounds were concentrated in a mid-similarity regime (0.322–0.731; median 0.478; IQR 0.435–0.524), consistent with thresholded analog retrieval. Functional-group profiling (Figure 2B) yielded counts of 3–13 (median 6; p90 10), with enrichment of hydroxyl/ether and carbonyl-related motifs. This pattern indicates that the retained set is chemically multifunctional rather than dominated by overly simple hydrophobic scaffolds.

In the top 20 subset (Figure 2C), pocket contacts were the main contributor to ranking (34–72; median 45.5), whereas hydrogen bonds were relatively sparse (0–4; median 1). Steric clashes ranged from 0 to 11 (median 4.5), and pocket_score spanned 33–53 (median 36). These data indicate that shape/surface complementarity dominated score separation under the current setup, with hydrogen-bond terms providing secondary refinement.

Joint evaluation of pocket_score and QED (Figure 2D) showed no meaningful correlation (Pearson r = 0.021), supporting Pareto-based multi-objective triage rather than forced early collapse into a single scalar metric. Metal electrostatic similarity was also weakly associated with total score (Figure 2E; Pearson *r* = −0.080), suggesting that, in the current weighting scheme, the metal term acted mainly as a modest modifier. Consistent with this finding, uncertainty in Ni^2+^ assignment is unlikely to be the dominant driver of final ranking; future studies will include Mg^2+^-only versus Mg^2+^+Ni^2+^ sensitivity analysis with explicit coordination-distance/angle monitoring.

### 3.3 Chemical-space compression using fingerprint clustering and tiered docking/energy screening

**Figure 3:**
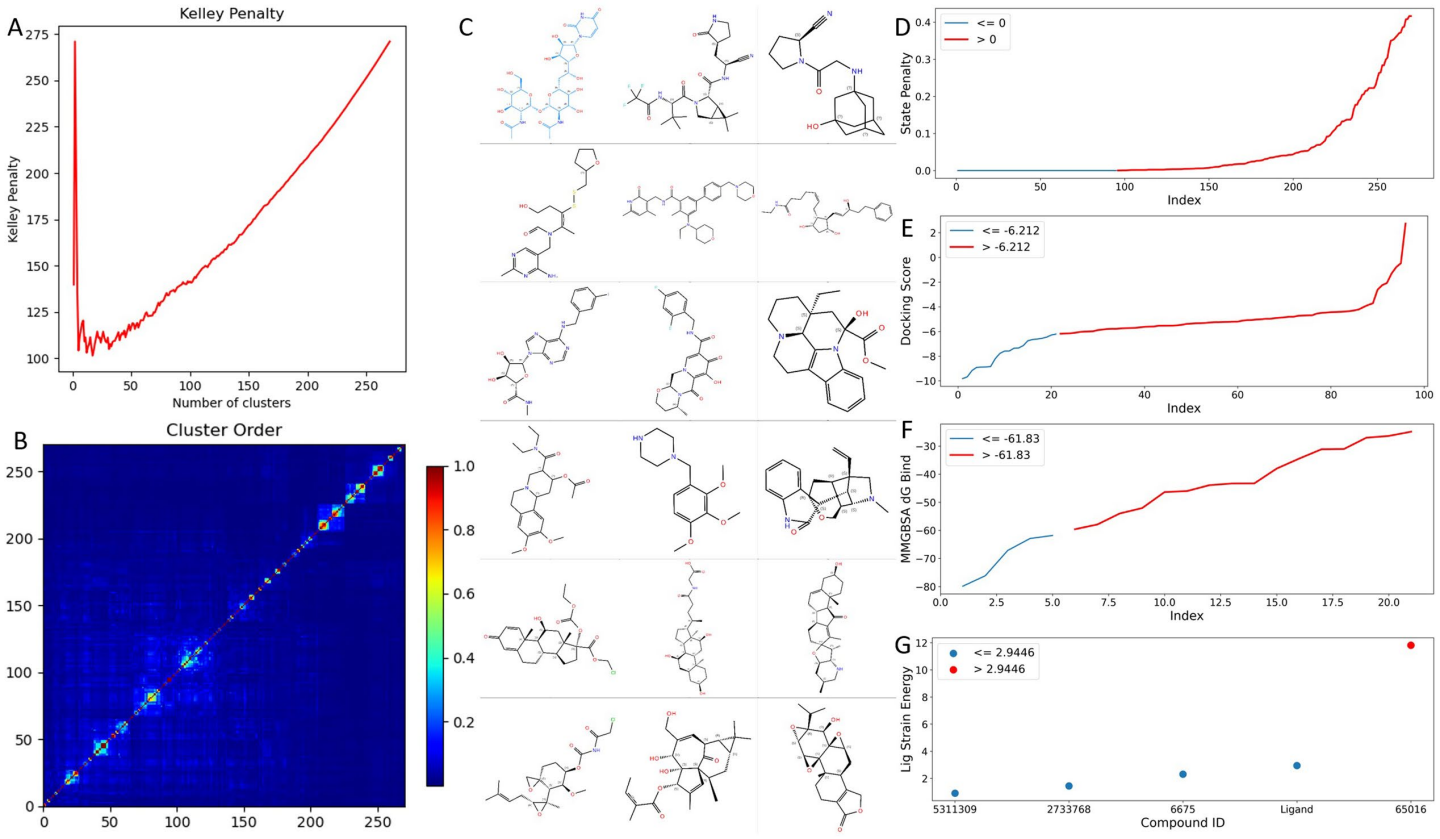
Fingerprint similarity clustering and tiered docking energy screening of 270 candidates. **(A)** Kelley penalty versus number of clusters from Canvas. **(B)** Canvas fingerprint similarity matrix 270 × 270 reordered by cluster membership. **(C)** Representative structures with 18 compounds, including 17 cluster representatives and the MRAY_PSEAE reference ligand. **(D)** State penalty distribution. **(E)** Docking score comparison with the reference ligand. **(F)** Prime MM-GBSA comparison with the reference ligand. **(G)** Ligand strain energy comparison.

To reduce redundancy while preserving representative coverage of the 270-compound set, we performed Canvas fingerprint-similarity hierarchical clustering (Figures 3A,B). The Kelley criterion selected 17 clusters with clustering strain = 1.263, and the reordered similarity matrix showed a clear diagonal block structure, indicating strong within-cluster similarity and low inter-cluster similarity in the fingerprint space. Figure 3C summarizes 17 cluster representatives together with the reference ligand. The full list of generated and selected compounds is provided in Supplementary Table S1.

Candidates were then filtered through a tiered structure-based workflow (Figures 3D–G). State-penalty filtering removed 175 compounds with state penalty > 0, retaining 95 candidates with more plausible protonation/tautomeric states under modeled conditions. Because DockingScore can include a state-related penalty contribution, this pre-filtering step reduced confounding due to low-probability ionization/tautomer states before rank comparison. At the docking stage, 21 candidates scored better than the reference ligand (reference DockingScore = −6.212; Figure 3E).

Prime MM-GBSA rescoring reduced this set to four candidates outperforming the reference baseline (reference MM-GBSA = −61.83 kcal/mol; Figure 3F). Ligand strain filtering further narrowed the shortlist to three compounds with lower bound-state strain than the reference (reference strain = 2.9446 kcal/mol): 6675, 2733768, and 5311309 (Figure 3G).

Because this is a charged, metal-dependent system, MM-GBSA values were interpreted strictly as comparative ranking signals under one consistent protocol rather than as absolute affinity estimates. Known limitations include approximate solvation/entropy treatment, sensitivity to protonation/ion assignment, and incomplete explicit treatment of metal-coordination energetics.

### 3.4 Binding-mode interpretation of prioritized hits and 1-μs MD stability assessment

**Figure 4:**
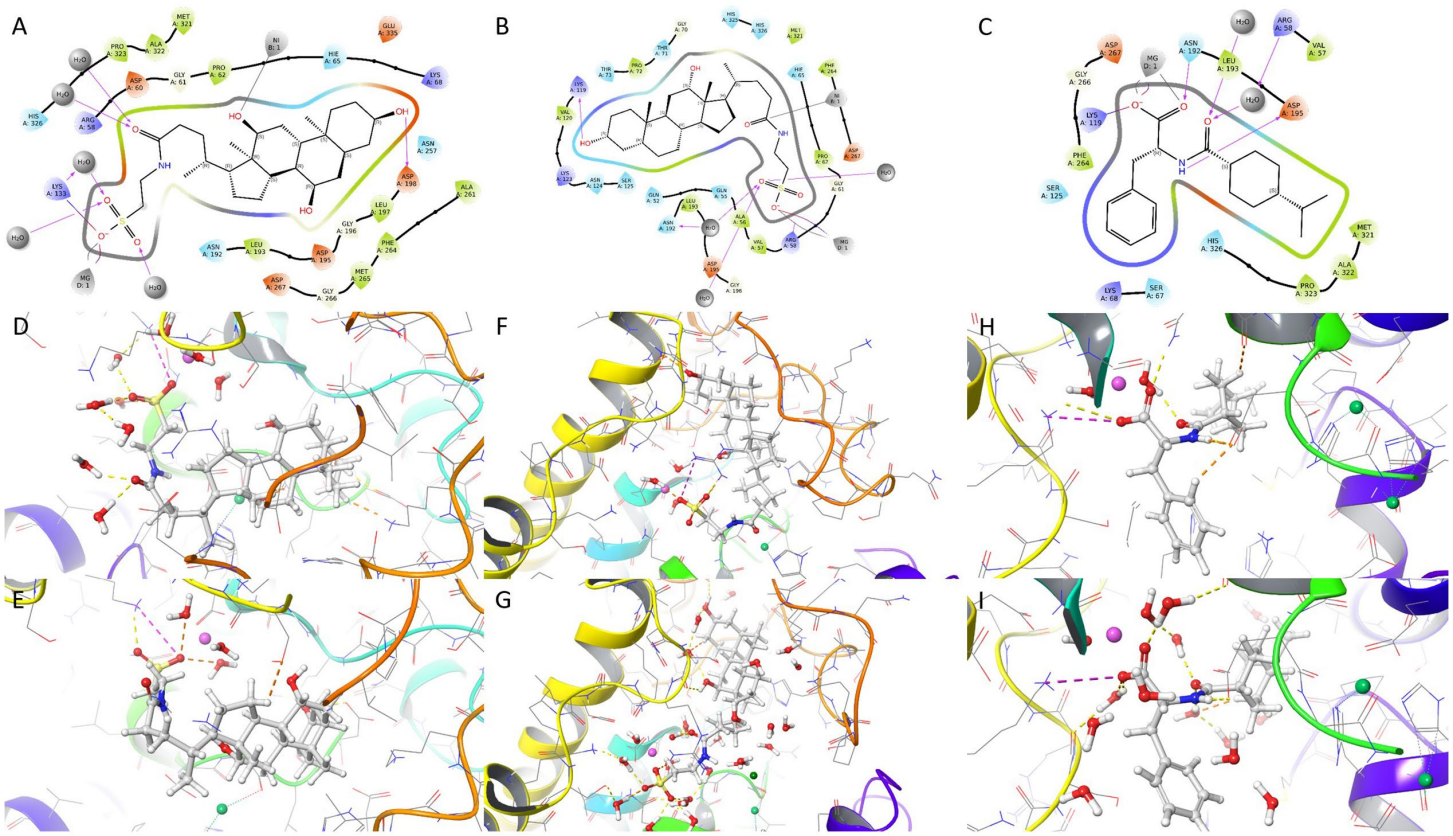
Binding mode analysis of prioritized hits and pose retention in 1.0 μs MD. **(A)** 2D interaction diagram of compound 6,675. **(B)** 2D interaction diagram of compound 2733768. **(C)** 2D interaction diagram of compound 5311309. **(D,E)** Representative 3D binding snapshots of 6,675 during 1.0 μs MD. **(F,G)** Representative 3D binding snapshots of 2733768 during 1.0 μs MD. **(H,I)** Representative 3D binding snapshots of compound 5311309 during 1.0 μs MD. Protein is shown as a cartoon ligand, waters as sticks, and metal ions as spheres. Dashed lines are direct or water-mediated contacts.

To rationalize the final shortlist, we examined interaction diagrams for 6675, 2733768, and 5311309 (Figures 4A–C) and then evaluated pose retention in 1-μs MD trajectories (Figures 4E–I). Compound 6,675 adopted a multi-anchor mode with polar contacts involving Arg58, Lys133, and Asp198, together with Mg^2+^/Ni^2+^-associated contacts and broad hydrophobic packing (including Leu193, Phe264, Met321, Ala322, and Pro323).

Compound 2733768 showed dual polar anchoring and strong core packing: a terminal hydroxyl–Lys119 interaction, a sulfonyl-centered polar/ionic network (including Ala56 and Arg58), a water-mediated support near Asn192, and metal-proximal contacts involving Mg^2+^ and Ni^2+^. The scaffold occupied a broad pocket region spanning both polar and hydrophobic subsites.

Compound 5311309 displayed a more focused carboxylate-centered anchor, with Lys119 ionic/H-bond support, Mg^2+^-proximal stabilization, and persistent polar reinforcement by Asn192, Leu193, and Asp195. Hydrophobic complementarity was concentrated near Phe264 and the Met321/Ala322/Pro323 flank.

Across 1-μs trajectories, compound 5311309 retained the most stable overall binding conformation, whereas 6675 and 2733768 exhibited larger conformational adjustments. The combined evidence from interaction and dynamics therefore prioritized compound 5311309 as the most stable binder among the three finalists under the current protocol.

### 3.5 Trajectory-level interaction hotspots and energetic stability of compound 5311309 versus the reference ligand

**Figure 5:**
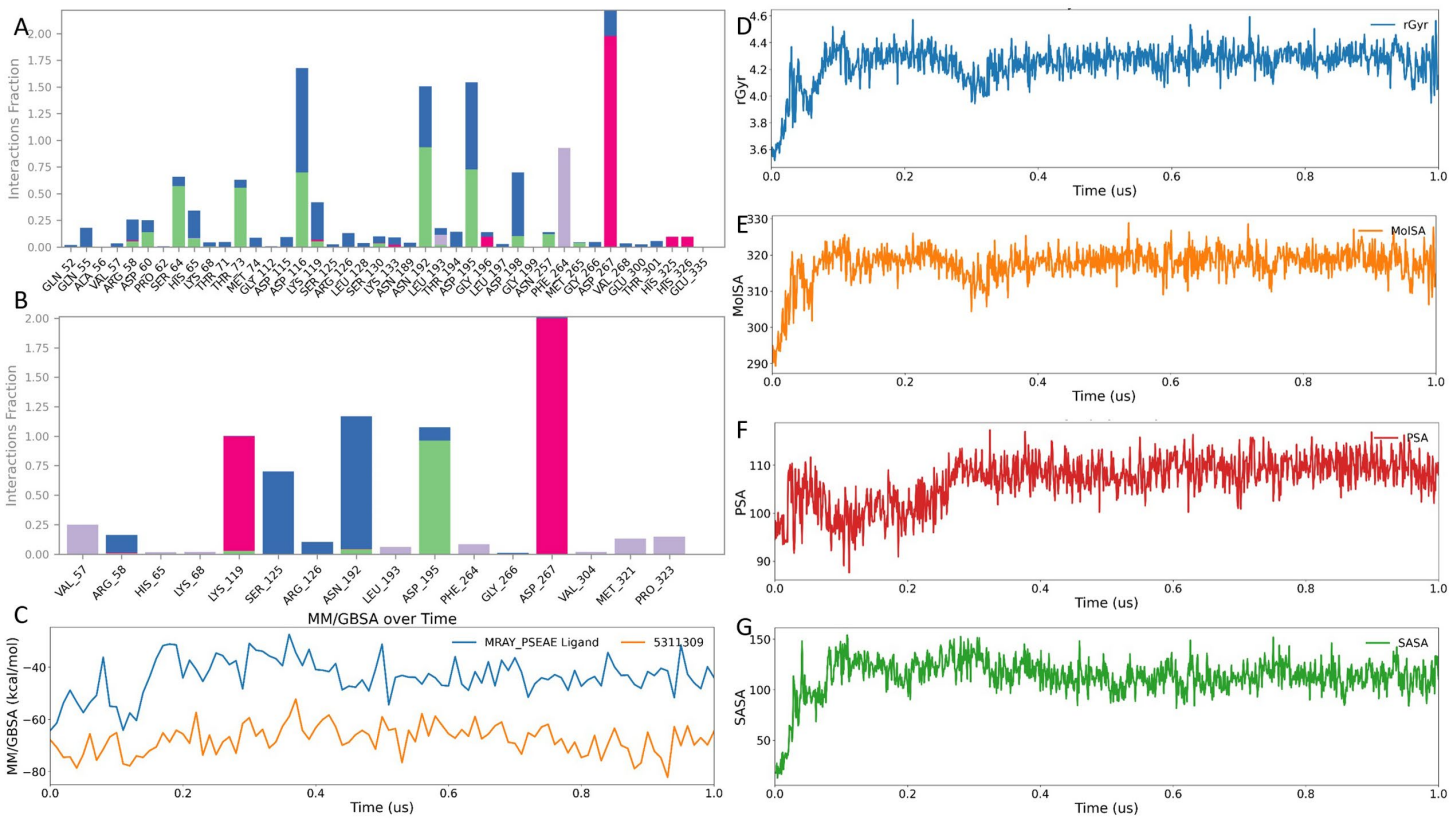
Trajectory-level interaction hotspots and stability of compound 5311309 versus the reference ligand over 1.0 μs. **(A)** Residue-wise interaction fraction profile for the reference ligand. **(B)** Residue-wise interaction fraction profile for compound 5311309. **(C)** MM-GBSA over time for the reference ligand and compound 5311309. *X* axis is time in μs, and *y* axis is MM-GBSA in kcal/mol. **(D)** Radius of gyration rGyr of compound 5311309 over time. **(E)** MolSA of compound 5311309 over time. **(F)** PSA of compound 5311309 over time. **(G)** SASA of compound 5311309 over time.

Using the trajectory-analysis workflow described in the section “Methods (Section 2.9),” we compared compound 5311309 with the reference ligand at residue resolution over 1 μs (Figures 5A,B). The two profiles shared dominant hotspots, indicating preservation of the same primary binding axis rather than site switching. Asp267 was the principal high-frequency node in both systems, with additional overlap at Asn192, Asp195, Ser125, Arg126, Arg58, and Val57. Recurrent hydrophobic engagement around Phe264 and retention of the Met321/Pro323 flank were also observed.

Despite this shared hotspot architecture, contact distribution differed. The reference ligand sampled a broader footprint across multiple pocket neighborhoods, whereas compound 5311309 showed a tighter interaction corridor from Lys119 via Ser125/Arg126 toward Asn192/Leu193/Asp195/Asp267, with limited peripheral sampling. This pattern is consistent with stronger focal anchoring and reduced exploratory motion.

Trajectory MM-GBSA profiles (Figure 5C) supported a more favorable energetic baseline for compound 5311309 throughout the full window. The reference ligand fluctuated approximately from −65 to −30 kcal/mol (most frequently −50 to −35), whereas compound 5311309 fluctuated from −82 to −55 kcal/mol (most frequently −72 to −60). Both traces showed local fluctuations without sustained drift, consistent with stable plateau-like sampling.

Ligand property trajectories for compound 5311309 (Figures 5D–G) showed early adjustment followed by stabilization: slight increases in the radius of gyration and MolSA, larger but bounded SASA fluctuations with an upward tendency, and early rise-then-plateau behavior of polar-surface metrics. Torsion analysis indicated that most rotatable bonds remained within narrow windows, with only one torsion showing notable early variability before settling.

### 3.6 *In silico* ADMET profiling suggests a permeability/efflux bottleneck with focused liver–kidney and irritation/sensitization flags

To assess developability alongside binding performance, we summarized model-predicted ADMET outputs and treated them strictly as hypothesis-generating signals. Overall, the profile suggests a meaningful exposure bottleneck driven by permeability and transporter effects, together with distribution and safety risks (Hakkola et al., 2020). In particular, predicted high plasma protein binding with low unbound fraction and hepatobiliary risk signals limit confidence in translational potential at this stage. Therefore, we interpret compound 5311309 as an early optimization hit rather than a development-ready Gram-negative antibacterial candidate. Detailed ADMET data are provided in Supplementary Table S2.

For absorption, Caco-2 = −5.649 and MDCK = −5.06 are both predicted as high risk (Bittermann and Goss, 2017), whereas PAMPA is predicted to be positive (Gousiadou et al., 2023). This Caco-2/MDCK versus PAMPA divergence is compatible with a transporter-mediated component but remains hypothetical without experimental validation. Consistently, the P-gp inhibitor is predicted negative, and the P-gp substrate is predicted positive, which may reduce intracellular exposure through efflux (Rehman et al., 2025). For Gram-negative antibacterial development, these liabilities are especially consequential because efficacy depends on sufficient intracellular accumulation despite permeability barriers and active efflux. Predicted bioavailability thresholds are mixed (F20% and F30% favorable; F50% unfavorable), and HIA is predicted to be acceptable; these outputs should be regarded as preliminary rather than definitive *in vivo* evidence.

For distribution, PPB = 98.1% and Fu = 2.7% are predicted and flagged as high risk, which may reduce free circulating and infection-site exposure. The predicted VDss = −0.878 may be compatible with relatively limited tissue distribution. Poor BBB penetration is predicted, which may be acceptable for non-CNS indications (McCartan et al., 2023). Transporter predictions are mixed: OATP1B1/OATP1B3 are not strongly flagged, whereas BCRP and BSEP inhibition are predicted (Anabtawi et al., 2022). The BSEP signal warrants specific caution because of potential cholestatic liability and therefore represents a key translational risk hypothesis requiring dedicated follow-up (Ren et al., 2021).

For metabolism and excretion, most CYP inhibitor calls are predicted negative, while CYP2C9 and CYP3A4 substrate calls are predicted positive. This pattern may suggest metabolism through these pathways and potential sensitivity to inducers/inhibitors. HLM stability is predicted to be favorable. Predicted plasma clearance (CL = 1.287) is not in a high-clearance range, and predicted half-life (T1/2 = 1.128, model-specific units) may be short to moderate. These outputs should be viewed as prioritization cues for PK experiments rather than quantitative PK conclusions.

For toxicity, several early screens are predicted to be favorable, including low hERG-related risk (hERG = 0.104; 10 μM = 0.073) and favorable AMES/carcinogenicity predictions (AMES = 0.084; carcinogenicity = 0.048). Multiple cytotoxicity readouts are also predicted to be favorable. However, higher-risk predictions are observed for human hepatotoxicity (0.767), DILI (0.499), nephrotoxicity (0.724), skin sensitization (0.857), and eye irritation (0.918), with additional flags for ototoxicity (0.706) and respiratory toxicity (0.561). A genotoxicity score of 0.479, together with a favorable AMES, should be treated as an uncertain mixed signal requiring orthogonal testing (Monem et al., 2025). These toxicity outputs further support cautious translational interpretation.

Taken together, these *in silico* outputs define two immediate optimization priorities: improving intracellular exposure by mitigating permeability/efflux liabilities, and early de-risking of hepatobiliary/renal and irritation/sensitization signals while preserving favorable predicted hERG and mutagenicity profiles. Accordingly, the current ADMET results are presented as model-based risk hypotheses, and compound 5311309 is positioned as a lead-optimization starting point rather than a clinically translatable Gram-negative antibacterial candidate at this stage.

### 3.7 Concentration-dependent growth inhibition defines distinct IC50 and MIC thresholds for compound 5311309 and the positive control Tunicamycin

Compound 5311309 showed clear concentration-dependent inhibition of bacterial growth, as measured by endpoint OD600. At concentrations up to 8 μM, growth remained close to the vehicle level, whereas a steep decline was observed across ~10–20 μM on the log concentration scale. Four-parameter dose–response fitting yielded an IC_50_ of approximately 15.1 μM. In the same assay, the positive control Tunicamycin (Catalog No. T13229) displayed substantially stronger low-dose activity, with an IC_50_ of approximately 0.30 μM, indicating higher apparent potency than compound 5311309 under these conditions.

**Figure 6:**
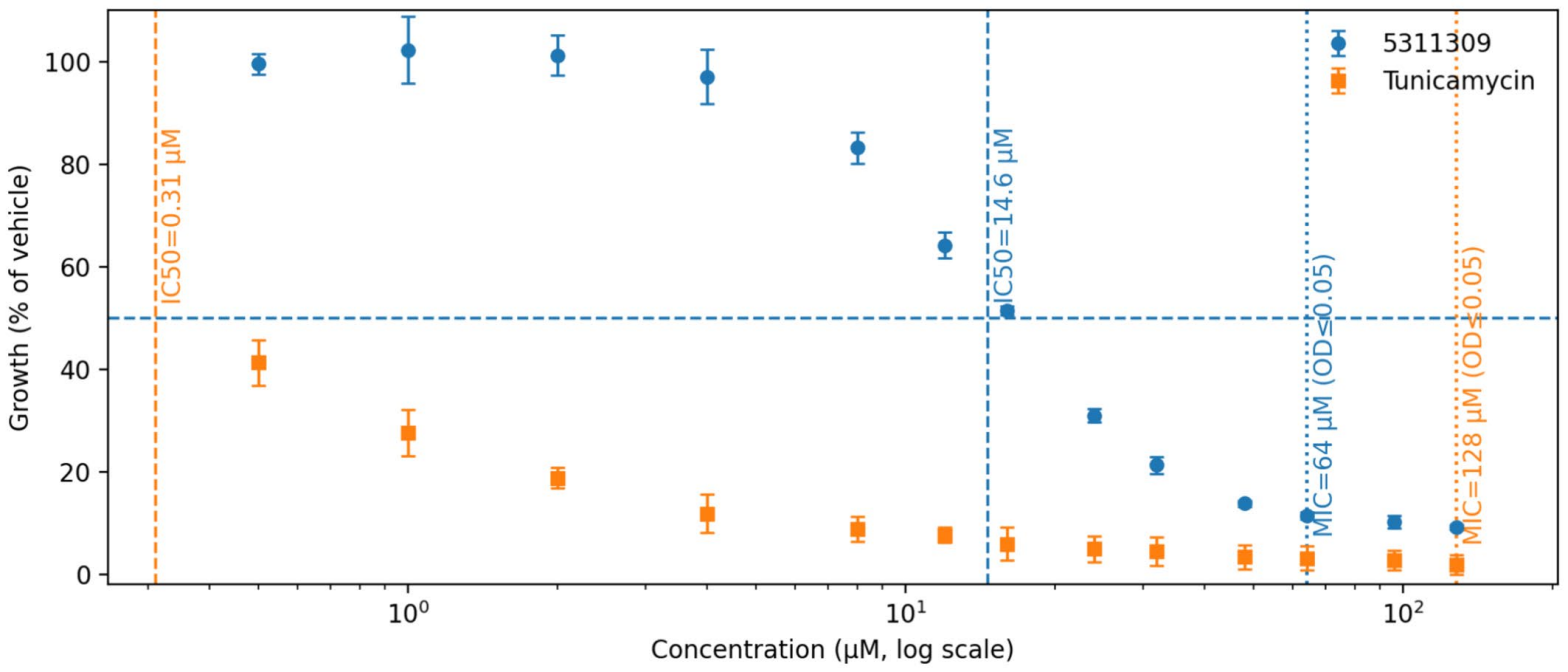
Dose–response growth inhibition of *Pseudomonas aeruginosa* by compound 5311309 and Tunicamycin.

With further dose escalation, OD_600_ values approached baseline. Using the operational criteria applied in this study (OD600 ≤ 0.05 with no detectable growth in 5/5 replicates), the MIC was 64 μM for compound 5311309 and 128 μM for Tunicamycin. The separation between IC_50_ and MIC indicates that partial growth suppression occurs at lower concentrations, whereas a stringent no-growth endpoint can be reached only at higher concentrations. These data support follow-up work to clarify whether the dominant effect is bacteriostatic or bactericidal and how the phenotype depends on exposure duration and inoculum level; mechanistic interpretation also remains preliminary because direct intracellular MraY target engagement has not yet been demonstrated, and off-target effects (e.g., membrane perturbation or other pathways) cannot currently be excluded (see Figure 6).

## 4 Conclusion

This study establishes a metal-aware, pocket-conditioned discovery workflow tailored to MRAY_PSEAE, where Mg^2+^-dependent catalysis imposes strict geometric and electrostatic constraints that can confound standard docking/scoring. By integrating explicit metal-site representation, chemical-space compression, tiered energetic rescoring (including MM-GBSA and strain-energy control), and microsecond-scale MD validation, we efficiently reduced 270 candidates to three prioritized hits and identified compound 5311309 as the most dynamically stable binder. The overlap of interaction hotspots between compound 5311309 and the reference ligand supports a conserved binding axis within the catalytic pocket. Functionally, compound 5311309 produced clear, dose-dependent growth inhibition with an IC50 in the mid-μM range (~15.1 μM) and a higher MIC threshold (64 μM), motivating follow-up mechanistic assays (target engagement and bacteriostatic/bactericidal resolution). *In silico* ADMET profiling highlights two immediate optimization axes: (i) improving intracellular exposure by mitigating permeability/efflux limitations and (ii) early de-risking of predicted hepatic/renal and irritation/sensitization liabilities while maintaining favorable hERG and mutagenicity predictions. Collectively, these findings provide both a methodological template for metal-dependent antibacterial targets and a concrete chemical starting point for anti-*P. aeruginosa* lead development. A key limitation is that this study does not implement diffusion/VAE/GAN-based *de novo* generation; instead, it explores a near-neighbor chemical space via similarity-guided library expansion, which improves practical tractability but may constrain scaffold novelty. A further limitation is the absence of formal retrospective benchmarking with known MraY inhibitors/decoys. Future studies will include such benchmarking and orthogonal experimental validation.

## Data Availability

The raw data supporting the conclusions of this article will be made available by the authors, without undue reservation.

## Footnotes

1
https://www.deshawresearch.com/
